# Piecing together the narrative of #longcovid: an unsupervised deep learning of 1,354,889 X (formerly Twitter) posts from 2020 to 2023

**DOI:** 10.3389/fpubh.2024.1491087

**Published:** 2024-12-16

**Authors:** Qin Xiang Ng, Liang En Wee, Yu Liang Lim, Rebecca Hui Shan Ong, Clarence Ong, Indumathi Venkatachalam, Tau Ming Liew

**Affiliations:** ^1^Saw Swee Hock School of Public Health, National University of Singapore and National University Health System, Singapore, Singapore; ^2^SingHealth Duke-NUS Global Health Institute, Duke-NUS Medical School, Singapore, Singapore; ^3^Department of Infectious Diseases, Singapore General Hospital, Singapore, Singapore; ^4^Department of Infection Prevention and Epidemiology, Singapore General Hospital, Singapore, Singapore; ^5^SingHealth Duke-NUS Medicine Academic Clinical Programme, Duke-NUS Medical School, Singapore, Singapore; ^6^Department of General Medicine, Tan Tock Seng Hospital, Singapore, Singapore; ^7^Health Services Research, Changi General Hospital, Singapore, Singapore; ^8^Department of Psychiatry, Singapore General Hospital, Singapore, Singapore

**Keywords:** long COVID, X, Twitter, social media, BERTopic, topic modeling, machine learning

## Abstract

**Objective:**

To characterize the public conversations around long COVID, as expressed through X (formerly Twitter) posts from May 2020 to April 2023.

**Methods:**

Using X as the data source, we extracted tweets containing #long-covid, #long_covid, or “long covid,” posted from May 2020 to April 2023. We then conducted an unsupervised deep learning analysis using Bidirectional Encoder Representations from Transformers (BERT). This method allowed us to process and analyze large-scale textual data, focusing on individual user tweets. We then employed BERT-based topic modeling, followed by reflexive thematic analysis to categorize and further refine tweets into coherent themes to interpret the overarching narratives within the long COVID discourse. In contrast to prior studies, the constructs framing our analyses were data driven as well as informed by the tenets of social constructivism.

**Results:**

Out of an initial dataset of 2,905,906 tweets, a total of 1,354,889 unique, English-language tweets from individual users were included in the final dataset for analysis. Three main themes were generated: (1) General discussions of long COVID, (2) Skepticism about long COVID, and (3) Adverse effects of long COVID on individuals. These themes highlighted various aspects, including public awareness, community support, misinformation, and personal experiences with long COVID. The analysis also revealed a stable temporal trend in the long COVID discussions from 2020 to 2023, indicating its sustained interest in public discourse.

**Conclusion:**

Social media, specifically X, helped in shaping public awareness and perception of long COVID, and the posts demonstrate a collective effort in community building and information sharing.

## 1 Introduction

The Coronavirus Disease 2019 (COVID-19) pandemic, which originated with the first reported case in China in December 2019 (1), has placed an immense burden on global health systems for the past few years (2, 3). While the initial COVID-19 infection predominantly manifests as a respiratory illness (4), a subset of individuals has reported enduring persistent and sometimes debilitating symptoms that extend beyond the acute phase (5). These symptoms, often referred to as long-haul or post-acute sequelae of SARS-CoV-2 infection (PASC), have drawn attention due to their potential to significantly impact the quality of life and functioning of those afflicted (6, 7).

A significant turning point in the understanding of long COVID occurred when the World Health Organization (WHO) provided an official definition for long COVID, defining it as the persistence or emergence of new COVID-19 symptoms 3 months following the initial COVID-19 infection, with these symptoms enduring for a minimum of 2 months and lacking any alternative explanation (8). This milestone signified a substantial shift in perspective, as long COVID had initially faced skepticism from some healthcare professionals and researchers (9, 10). It is essential to acknowledge that individuals with long COVID have reported experiencing dismissal or a sense of ‘gaslighting’ from medical staff (10, 11). Long COVID has since garnered increased recognition among the medical and scientific community, leading to the development of clinical management guidelines for long COVID-19. This progress includes the introduction of European Society of Clinical Microbiology and Infectious Diseases (ESCMID) guidelines for the management of long COVID, and the refinement of the National Institute for Health and Care Excellence (NICE) guidelines for addressing the long-term effects of COVID (12–15). Researchers have observed that long COVID appears to have a higher prevalence among females, individuals with obesity, those with pre-existing comorbidities, and older age cohorts, and is associated with significant indirect and direct costs (16).

## 2 Related works

Tracing the roots of long COVID, one would quickly recognize that in May 2020, Dr. Elisa Perego first introduced the hashtag “#longcovid” on X (formerly Twitter), originally using it to describe her own experience of enduring “cyclical, multiphasic, and multisystem” COVID-19 symptoms (17). The hashtag rapidly gained prominence, transforming into a virtual space where individuals grappling with persistent COVID-19 symptoms could connect, exchange experiences, and voice concerns. This rapid dissemination on X (formerly Twitter) not only gave a voice to those living with long COVID but also demonstrated the substantial influence social media platforms wield in social mobilization and shaping global health discussions (18–20). Specific to long COVID, four previous studies exist (21–24). Turner et al. conducted a manual reflexive thematic analysis of tweets containing #longcovid from May 20 through August 21, 2020. The study highlighted a significant degree of social constructionism, with X (formerly Twitter) users contributing to the awareness of long COVID. They achieved this by collectively agreeing on the symptoms of long COVID, potentially leading to greater recognition and enhanced healthcare services for sufferers (21). Santarossa et al. investigated the usage of #longcovid and #longhauler hashtags from February 18 to February 23, 2021. They discovered that in discussions tagged #longcovid, the terms ‘support’ and ‘research’ were mentioned in 56.5 and 22.5% of the tweets, respectively. In the case of #longhauler, ‘symptoms’ and ‘building a community’ were prominent in 61.5 and 31.5% of the discussions, respectively (22). Awoyemi et al. also performed a sentiment analysis on long COVID mentions on X (formerly Twitter), extracting key phrases and analyzing 10,670 tweets from March 25 to April 1, 2022, spanning a week. Their study found that sentiments on X (formerly Twitter) about long COVID were almost evenly divided, with 19.9% positive and 18.4% negative reactions (23). A newer study by Kusuma and Suherman, however, extracted tweets containing the keyword “long COVID” from December 1, 2022, to February 22, 2023, and found that most tweets (49%) were negative, reflecting significant concern and frustration (24). To fully evaluate the data over time, a longer analysis period and newer data might be necessary. Despite the advent of COVID-19 vaccines and therapeutics, people still remain vulnerable to long COVID and its effects. Much is also still unknown about long COVID and its impact on people over time (25, 26). Moreover, compared to the study by Kusuma and Suherman, which used social network analysis (SNA) and sentiment analysis, focusing on identifying influencers and mapping the social network structure (24), this study would delve into the content of the tweets using Bidirectional Encoder Representations from Transformers (BERT)-based unsupervised topic modeling. This would enable a more granular exploration of themes within the discourse rather than just identifying influential nodes in the network; the voices of the long COVID community could foster increased awareness, knowledge dissemination and enhanced health services for individuals grappling with long COVID.

X (formerly Twitter) serves as a rich data source for real-time discourse, enabling the tracking of public opinion on health topics of significant public interest. Previous studies have demonstrated its value in understanding public health trends and discourse (21–24). An analysis of long COVID sentiments on X (formerly Twitter) holds the potential to provide researchers and healthcare professionals with a qualitative understanding of the challenges, emotions, and concerns expressed by those affected by long COVID, thereby contributing to informed views and enhanced patient care. A two-year follow-up study has revealed that a majority of individuals continue to face a heightened risk for conditions associated with long COVID, such as diabetes, respiratory issues, chronic fatigue, and blood dyscrasias, among other conditions impacting the gastrointestinal and musculoskeletal systems (27). In line with these findings, an online survey study found that long COVID manifestations, particularly neuropsychiatric symptoms, persist at 6, 12, and 24 months post-COVID-19 infection (28). Moreover, the risk of developing long COVID appears to be relatively unaffected by one’s vaccination status (29). This makes long COVID still very much relevant today despite the billions of doses of COVID-19 vaccines deployed and the endemic status of COVID-19.

Prior studies have observed that COVID-19 discussions on X (formerly Twitter) undergo temporal shifts over time (29, 30), suggesting that incorporating a temporal dynamic could provide clearer understandings of the discourse surrounding issues of public concern. This approach can yield valuable insights into how the public perceives long COVID vis-à-vis major COVID-19 events, such as the emergency authorization of vaccines and emergence of new COVID-19 variants. By observing these fluctuations and interpreting them in the context of significant events and governmental decisions, we can better anticipate the potential impact of future similar measures.

Our study thus aims to analyze tweets collected over a three-year period to explore the themes, public opinions, and sentiments regarding long COVID. Additionally, we seek to examine trends and shifts in these aspects in relation to major COVID-19 milestones in the United States (US), where most X (formerly Twitter) users reside (31). We hypothesize that there would be shifts in public discourse following key milestones in the COVID-19 timeline, e.g., the US Food and Drug Administration (FDA)’s Emergency Use Authorization of the Pfizer/Comirnaty vaccine and the emergence of new variants of concern (VOC), e.g., the Delta and the Omicron variants. From a social constructivist perspective (32, 33), public discourse on social media is not merely a reflection of individual opinions but a socially constructed narrative, shaped by influential figures, events and other sources. Through this research, we seek to better understand the public perceptions and conversations surrounding long COVID and its evolution over time.

## 3 Methodology

The research methodology employed in this study was inspired by earlier infodemiology research that similarly used X (formerly Twitter) to explore public sentiment and emotions on specific topics (20–24). In this study, X (formerly Twitter) served as the primary social media platform, with the search queries #longcovid, #long_covid, and “long covid” employed to gather English-language tweets. The collection period extended from May 20, 2020, marking the date of Perego’s first tweet with #longcovid, to April 1, 2023. Data retrieval was performed through X (formerly Twitter)’s Application Programming Interface (API) under an academic developer account, which allowed downloads up to 10 million tweets monthly without sampling. We excluded retweets, duplicates, and tweets from organizational accounts in our analysis. The study encompassed tweets globally, without geographical limitations on the origin of the tweets.

For processing the extensive textual data from X (formerly Twitter), we utilized the aforementioned BERT technology. BERT, developed by Google in 2018 and incorporated into Google’s search engine for English queries, represents a leading approach in natural language processing or NLP (33, 34). It uniquely processes text in both directions, considering the context of words within sentences. BERT’s effectiveness stems from its pre-training on vast amounts of text within a Transformer-based neural network, using masked language modeling where it predicts words masked in a sentence based on their context. Unlike traditional models, BERT does not require extensive text pre-processing like stemming or tokenization.

In identifying individual and organizational users, we applied BERT’s Named Entity Recognition (NER) feature, which discerns entities in text based on token sequences (35). BERT NER recognizes four entity types: location, organization, person, and miscellaneous. As X (formerly Twitter) dataset has two separate columns (i.e., free text for tweets, and a separate column for user names), BERT NER is applied to the “user name” column, assuming that user accounts typically use names that reflect individual identities for personal accounts and organizational identities for non-personal accounts. For this study, we included tweets identified as personal (PER) through BERT. In a previous evaluation on various datasets, BERT achieved a precision of 90.7%, a recall of 91.9%, and an F1-score of 91.3% (36). We also employed BERT-based topic modeling to derive key themes from public discussions (37). This unsupervised machine learning technique discovers patterns and clusters similar concepts within text documents, akin to traditional thematic analysis but automated for handling large datasets and helps to generate interpretable topics (38). The topics identified through topic modeling were manually reviewed and labeled by our researchers, who also grouped them into overarching themes using Braun and Clarke’s inductive thematic analysis approach (39). This iterative process involved four authors, with backgrounds in medicine, social science and psychology, and it entailed familiarization with the data, preliminary coding, theme formulation, refinement, and final definition, ensuring a thorough and nuanced analysis.

The constructs framing our analyses were data driven as well as informed by social constructivism, which posits that our knowledge and understanding are constructed through social interactions and processes (32, 33). It emphasizes how language, communication, and societal practices influence our perception of reality. Through this lens, the posts and narratives surrounding long COVID on platforms like X (formerly Twitter) represent a form of social construction, where individuals collectively navigate and construct the significance, understanding, and consequences of long COVID.

The study had ethical approval from the SingHealth Centralized Institutional Review Board of Singapore (reference no. 2021/2717), and complied with X (formerly Twitter)’s terms of use. No human participants were directly involved in this data collection. We also adhered to ethical guidelines outlined by the Association of Internet Researchers (AIOR) for reporting tweets and analyzing data from online communities (40).

## 4 Results

### 4.1 Retrieval of relevant posts

**Figure 1:**
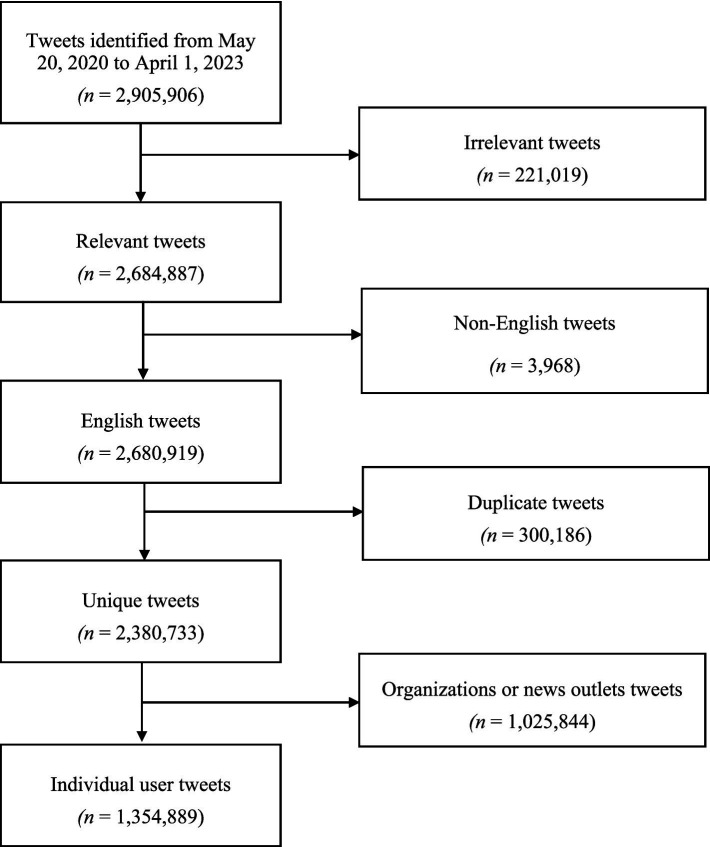
Flowchart showing the tweet extraction and selection process.

Out of an initial dataset of 2,905,906 tweets, a total of 2,380,733 unique English-language tweets were selected. Within this dataset, 1,025,844 tweets were identified as originating from organizations over X (formerly Twitter), while 1,354,889 unique tweets from individual users were included in the final dataset for analysis. Figure 1 provides a flowchart showing the tweet selection process.

**Figure 2:**
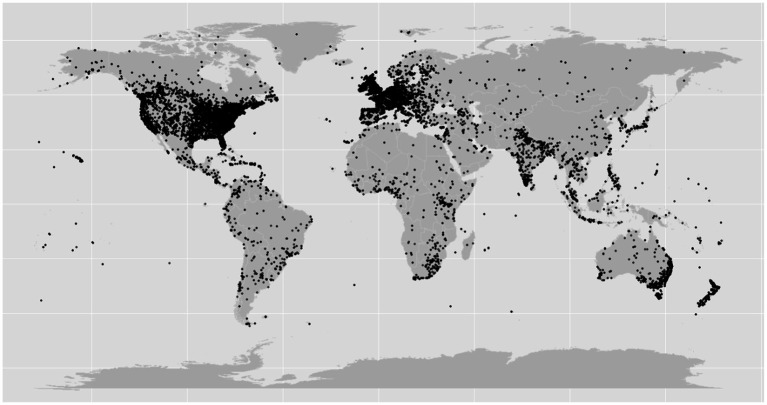
Geographical locations of the tweets included in this study (each tweet is indicated by a black dot in the map).

Analysis of the geolocation of the tweets found that the majority of these tweets originate from North America (28.7%), Europe (23.3%), Australia (4.7%) and Asia (1.5%), in alignment with the general demographic of English-speaking users over X (Figure 2) (31).

### 4.2 Topics based on topic modeling

With the use of unsupervised machine learning, the BERTopic model produced a total of 11 distinct topics (Table 1). Together, these topics accounted for 73.7% of the analysis, corresponding to 1,000,556 tweets. The other 26.3% of tweets were categorized under a ‘Miscellaneous’ topic by the BERT NLP model. This category included tweets that did not fit well with any of the identified topics. The model flagged these as outliers, effectively minimizing extraneous data that could otherwise skew the representation of the main topics.

**Table 1:** Themes related to the public discussions around long COVID, along with their respective topics and sample tweets (*N* = 1,354,889).

Theme and topic label (keywords)	Sample tweets	Number of tweets, *n* (%)
Theme 1: General discussions of long COVID		
Topic 1: Reports on long COVID (*deaths, die, infected government, dying, syndrome, dead, media, covid cases*)	“34,000 Canadian, the U.S. is on track for 1,000,000 Covid19 deaths. Viruses evolve evade vacs. What is your Covid plan to prevent further long COVID and deaths.” “Damn. Long COVID is scary!”	570,508 (42.1)
Topic 2: Raising awareness about long COVID (*longcovid patients, pwme, longcovid, longcovidkids, longcovid mecfs, mecfs longcovid, exercise, longcovid symptoms, sharing, longhaulers*)	“You are not alone. #pwME #LongCovid #doctorswithME” “The podcast is out today; a huge thank you to my guest grab a cuppa and have a listen #LongCovid #LongCovidKids #MECFS #POTS #TreatLongCovid”	128,188 (9.5)
Topic 5: Importance of wearing masks as a prevention strategy from long COVID (*mask, masks, wearing, wear, masking, wear mask, wearing mask, n95, wearing masks, mandates*)	“Sane Americans who do NOT want to get covid, or long covid, best mask up again. Sane Americans know that masking is easier than getting sick!” “The ability to contract it again and the increasing evidence of the shitshow of ongoing symptoms from long COVID? If you expect people to respect your decision not to wear a mask, you should be respectful the decision of others to wear one or request one be worn in their presence”	31,765 (2.3)
Topic 6: Public figures and COVID-19 (*trump, boris, guy, die, deaths, kaine, johnson, gets long*)	“You can bet he’ll be fine because healthcare in this country is always available if you can afford it. He will purchase antivirals and then tell us all how mild it was. Ignoring the hospital crisis gripping the country and the thousands with long COVID. More gaslighting” “He was anti-Vax and caught covid. Now he has long Covid. It’s not the common code and there’s reason to panic.”	24,938 (1.8)
Topic 9: Risk of long COVID with new variants of SARS-CoV-2 (*omicron, covid omicron, long COVID omicron, delta,* var*iant, variants, omicron variant, omicron mild, infected, immunity*)	“Approx 1.3 million Canadians estimated to have Long COVID from Omicron. Mild my ass.” “And then there’s #LongCovid I did not see it mentioned an only news channel that, despite a statistically lower risk from Omicron than Delta there were more people with LC from Omicron than Delta because of the number of cases! Yet new variant is recombined Delta and Omicron!”	11,505 (0.8)
Theme 2: Skepticism about long COVID		
Topic 3: Attributing long COVID to COVID-19 vaccines (*vaccines, unvaccinated, vaxxed, covid vaccine, immunity, long COVID vaccine, reduce, injury, covid vaccines, covid vaccinated*)	“I got long covid, still have but from the vaccine” “Long COVID = vaccine injury”	121,144 (8.9)
Topic 10: Denying the existence of long COVID (woman, vaxxed, vaccines, she, mask, lorenz, thinks, covid, blocked)	“Most of these cases are Vaccine Side Effects do your proper research And Sylvana CLAIMS to have Long COVID … so she can lay in her bed watch Netflix while Dutch Blacks are Starving” “Long COVID has been grossly over estimated. She needs to read up to date journals. Or stick a mask on and lie down somewhere quiet…give the rest of us a bit of peace”	10,701 (0.8)
Theme 3: Adverse effects of long COVID on individuals		
Topic 4: Long term effects of long COVID in children (*schools, covid children, parents, teachers, covid kids, long covid children, child, long COVID kids, children long, children long covid*)	“11–15% of children will experience some form of long COVID that’s 5,500–7,500 kids in Texas alone who are likely to experience potentially permanent symptoms who knows how many more will have invisible organ damage that will impact them in the future” “10 children died between 23rd Aug-26th Sept. 96 in total during the pandemic. Latest studies show healthy children are just as vulnerable. Then there’s the long term risks, e.g., Long Covid, diabetes, brain damage (Biobank study), early onset dementia…”	61,713 (4.6)
Topic 7: Work absenteeism due to long COVID (*workers, workforce, labor, jobs, staff, shortage, disabled, force, economy, people work*)	“Yep. And those affected early with long COVID are being laid off now, with no extra financial help, just the below par minimum, so shoved into poverty” “Long COVID is keeping as many as 4 million people out of work, according to research”	16,236 (1.2)
Topic 8: Cognitive issues associated with long COVID (*cognitive, covid brain, long COVID brain, neurological, memory, brain damage, covid brain fog, fog long covid, dementia, fog long*)	“All due to LONG COVID - BRAIN FOG?” “Its funny how covid twitter is arguing about how valid the brain imaging Biobank study is as if its the only evidence of effect on brain. People with #LongCovid have been describing neuro symptoms for ages with multiple studies to characterize them. People before tests, folks!”	12,360 (0.9)
Topic 11: Relationship between long COVID and chronic fatigue syndrome (*cfs, covid cfs, long COVID cfs, cfs long, cfs long covid, people cfs, cfs patients, decades, cfs, fibro*)	“I read the exact opposite. The most severe cases of long COVID develop ME/CFS, and there were several people with ME/CFS who’d had cancer before who said ME/CFS is much more debilitating.” “A small % of #LongCovid, as with any serious virus, may develop ME. Still q. early to tell - although a small no. of those who exercised through Covids PVF have been presenting w. CFS. COVID-19 is unique - crucially it’s all taking longer (so talk of ME still a bit previous).”	11,498 (0.8)

### 4.3 Reflexive thematic analysis

Through qualitative thematic analysis, we organized the topics into three main themes. These themes are as follows: Theme 1: General discussions of long COVID (encompassing Topics 1, 2, 5, 6 and 9), Theme 2: Skepticism about long COVID (comprising Topics 2 and 10), and Theme 3: Adverse effects of long COVID on individuals (comprising Topics 4, 7, 8 and 11). Table 1 contains details regarding the individual topics, key words and sample tweets within each theme.

Theme 1 captured the broad and overarching conversations about long COVID, reflecting public awareness and concerns. There were discussions about the severity and impact of long COVID, including references to deaths and government responses (Topic 1). Tweets also often mentioned high numbers of COVID-19 deaths and the evolution of the virus (Topic 1). Users also shared personal stories and support for those suffering from long COVID, often using hashtags like #longcovid, #MECFS (Myalgic Encephalomyelitis/Chronic Fatigue Syndrome), and #POTS (Postural Orthostatic Tachycardia Syndrome) (Topic 2). These tweets serve to create a community of support and raise awareness about the condition. There were also discussions about the importance of masking as a means to prevent COVID-19 and its long-term consequences (Topic 5). Discussions also focused on public figures such as Donald Trump and Boris Johnson who have contracted COVID-19 (Topic 6). Finally, there were also conversations around the risk of developing long COVID from newer variants like Omicron and Delta (Topic 9). Tweets expressed concern about the perceived higher incidence of long COVID despite the perceived mildness of some variants.

Under Theme 2, views ranged from skepticism to extreme conspiracy theories surrounding long COVID. Users have suggested that long COVID symptoms are actually vaccine injuries (Topic 3), reflecting a distrust in COVID-19 vaccines. There were also users who expressed disbelief in the existence of long COVID, sometimes attributing reported cases to other causes or dismissing them outright (Topic 10).

Finally, Theme 3 encompassed the personal and societal impacts of long COVID. There were tweets centered around the impact of long COVID on school-going children, including increased absenteeism and potential deleterious long-term health issues (Topic 4). Tweets also discussed the economic and labor implications of long COVID, highlighting how it leads to staffing shortages, workforce challenges and job losses (Topic 7). There were also tweets highlighting the cognitive issues associated with long COVID, such as brain fog and memory deficits (Topic 8). Some have also made the link between long COVID and chronic fatigue syndrome, exploring similarities in symptoms and impacts on health (Topic 11).

### 4.4 Analysis of temporal trends

The analysis of temporal trends (Supplementary Figure S1) showed that the topics and discussions around long COVID have remained relatively stable throughout the pandemic years, from 2020 to 2023. Long COVID has likely remained a topic of ongoing public interest or concern and has neither significantly diminished in relevance nor escalated drastically in the public eye.

**Figure 3:**
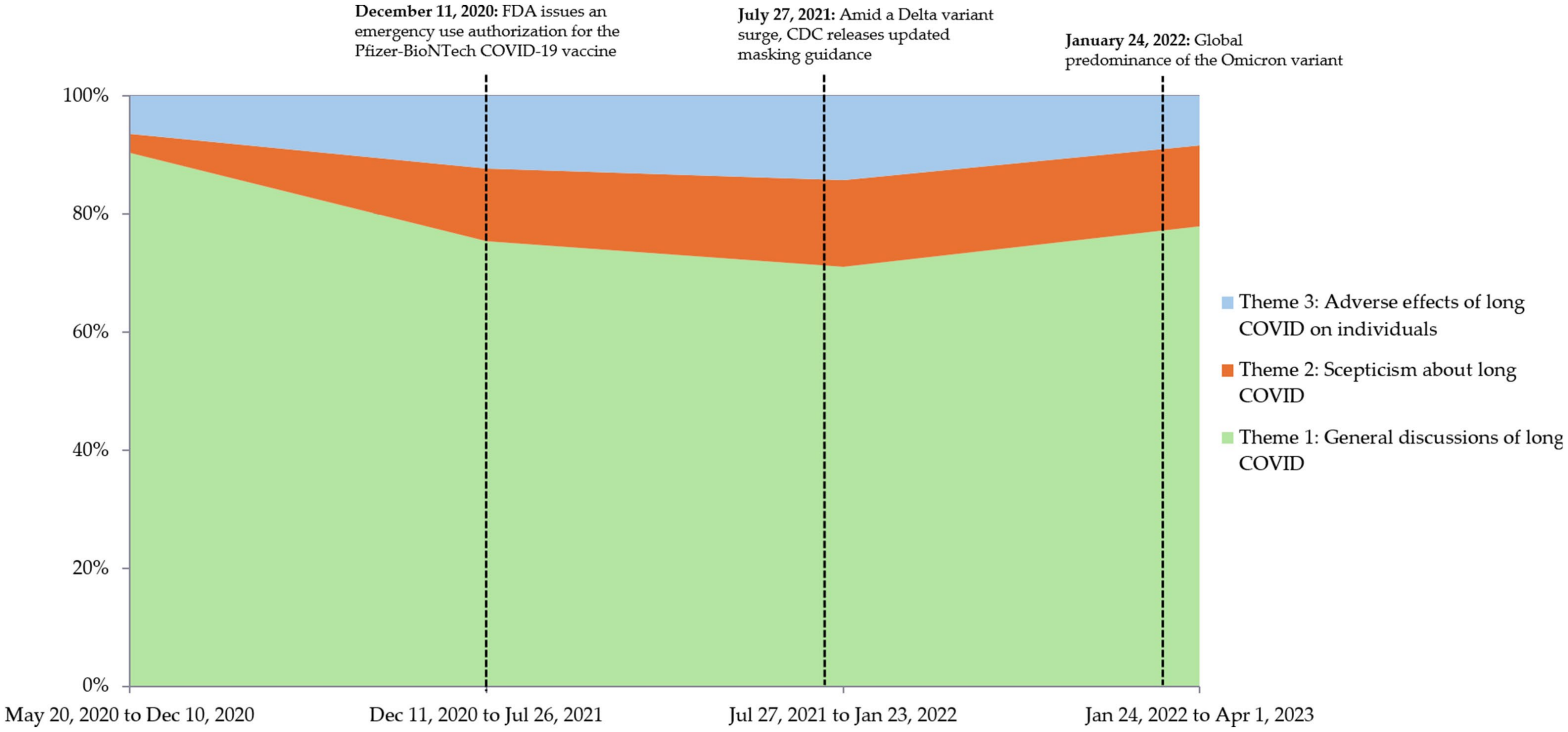
Trends in tweets belonging to Theme 1, 2, and 3 in relation to major milestones; dates taken with reference from the US Centers for Disease Control and Prevention (CDC) (40).

Further analysis of the temporal changes in these themes in relation to major milestones, e.g., US FDA’s EUA of the Pfizer/Comirnaty COVID-19 vaccine and emergence of new VOCs, was shown in Figure 3. Theme 1 captures the broad discussions about the general awareness, concerns, and impacts of long COVID. Before the FDA’s EUA of the COVID-19 vaccine, this theme was highly prominent as public interest and concern about long COVID were strong. There was a notable decrease after the FDA’s EUA: After the EUA, the prominence of this theme decreased. This could be attributed to the shift in public discourse surrounding vaccine-related topics and possibly due to the emerging narratives that either subsumed or reframed these general discussions within the context of vaccination and its impacts. Theme 2 encompassing skepticism about long COVID became more prominent after the FDA’s EUA and could be linked to the growing vaccine rollout, sparking debates, and skepticism about both the vaccine and long COVID. This theme persisted as discussions continued to revolve around vaccine efficacy and the legitimacy of long COVID. Theme 3, which talked about the adverse effects of long COVID on individuals, surprisingly increased after FDA’s EUA but declined during the large Omicron variant surge globally. The increased prominence of Theme 3 after the US FDA’s EUA might be due to a heightened awareness and reporting of long COVID symptoms as more people got vaccinated and started experiencing or reporting long COVID symptoms, while the decline of this theme during the Omicron surge could be due to the overwhelming focus on the new variant, its characteristics, and immediate impacts. The public discourse might have shifted to more immediate concerns about the spread and severity of Omicron, leading to a temporary dip in discussions about the long-term impacts of long COVID.

## 5 Discussion

Our analysis of 1,354,889 tweets from 2020 to 2023 offers a comprehensive overview of public sentiment and discourse on long COVID, a significant and persistent aftermath of the COVID-19 pandemic. The data, derived from X (formerly Twitter) using advanced NLP tools, reveals a complex landscape of public awareness, skepticism, and personal impact narratives regarding long COVID. In these spaces, individuals collectively navigate and construct the significance, understanding, and consequences of long COVID. Here, we discuss the implications of these findings and their relevance to public health, policy, and research.

The prevalence of general discussions about long COVID underscores the critical role of social media in raising awareness and fostering community support. Tweets in Theme 1 indicate a collective effort in sharing personal experiences, information, and support resources. This theme reflects a public consciousness about long COVID’s severity and impact, aligning with literature that emphasizes the role of social media in health communication and community building during pandemics (41, 42). Similar to the thematic analysis conducted by Turner et al. there is significant social constructionism, with X (formerly Twitter) users contributing to the awareness of long COVID and supplementing the possible constellation of symptoms of long COVID through shared beliefs, language and meaning (21, 38, 39). As X (formerly Twitter) is often a channel for people to share and seek health information, it is also unsurprising that tweets under Theme 3 covered personal anecdotes regarding the adverse effects of long COVID on individuals, including children and the workforce. The mention of cognitive issues and parallels drawn with chronic fatigue syndrome illustrate the multifaceted and enduring nature of long COVID as demonstrated in current research (28). This theme also aligns with current research on long COVID, which emphasizes the need for long-term care strategies and policy adjustments to accommodate individuals experiencing prolonged symptoms (43, 44).

On the flipside, there is also considerable public health harm that can result from X (formerly Twitter). Various tweets containing skepticism and conspiracy theories form part of the prevailing public discourse. The attribution of long COVID symptoms to vaccine injuries reflects prevalent misinformation and distrust in public health interventions. This misconception regarding vaccine injury has also been highlighted in previous studies on vaccine misinformation and hesitancy (45, 46). This highlights the need for continued public health education and transparent communication strategies to counteract misinformation, particularly in the era of vaccine hesitancy and misinformation (20). Of note, the prominence of Theme 2 after FDA’s EUA could be tied to the broader context of vaccine hesitancy. This hesitancy is influenced by debates and skepticism regarding vaccine efficacy and safety, as well as the legitimacy of long COVID, all of which are amplified and sustained by social media dynamics. Nonetheless, while the data highlight the evolution of public discourse, it is important to acknowledge that the study captures associations rather than causal links between online discussions and broader societal perceptions.

Given the general stability in temporal trends of long COVID discussions over 3 years, long COVID is evidently a topic of enduring relevance and importance in public discourse. This persistent attention suggests that long COVID remains a public health issue of concern, warranting continued research and healthcare resource allocation. Research today suggests that being vaccinated may in fact help to protect against the development of long COVID (25, 26), although the studies had high heterogeneity (47). Individuals also continue to be afflicted by the effects of long COVID and would require primary care services, rehabilitation services and continued patient support networks (48). In a recent study conducted in the UK, it was found that a diagnosis of long COVID was associated with a 43% increase in primary care consultation costs, compared with patients without long COVID symptoms (49). Unfortunately, despite public interest and significance, long COVID and related chronic conditions, where infections lead to persistent symptoms lasting months, years, or indefinitely, remain insufficiently researched and funded (50). The field has also become stagnant; currently, there are no recognized diagnostic methods or biomarkers for long COVID and studies often use divergent definitions, notwithstanding efforts by the WHO to harmonize the definition of long COVID (8, 51, 52). There is also no approved treatments for long COVID despite the consensus that this condition causes significant debilitation and suffering (25, 26). To move the field forward, it would be critical to bring together multidisciplinary groups working on long COVID as the effects span multiple organ-systems and have both physical and psychological dimensions.

The findings and themes identified in our study echo a holistic approach to the research and management of long COVID, encompassing not only medical aspects but also psychosocial support and public education. Policymakers and healthcare providers should acknowledge the diverse and agonizing experiences and concerns of those affected by long COVID, facilitating patient-centered care and support systems.

### 5.1 Study limitations

While our study provides valuable insights, it is not without limitations. First, despite the large dataset comprising more than a million unique, individual user tweets, we cannot assert with absolute certainty that our analysis achieved saturation given the open nature of the platform. The richness and variety of the topics might benefit from more targeted inquiries or supplementary qualitative methods in future studies. Second, the analysis is restricted to English-language tweets and may not fully capture the global diversity of experiences and opinions on long COVID, and Asian countries are noticeably underrepresented in the study sample. Third, topic modeling may not capture the complexity of human emotions, especially in nuanced or mixed-emotion contexts. Fourth, the tweets examined in this study are self-reported and not verified for accuracy. There is no distinction between tweets from individuals with medically confirmed long COVID and those speculating or sharing second-hand information. Hence, some interpretations may be erroneous as a result. Fifth, our data collection concluded on April 1, 2023, in response to changes in X (formerly Twitter)’s policy and limitations on data download. This external constraint affected our ability to continue collecting tweets beyond this date. Sixth, our focus was solely on the term ‘long COVID’ and did not include alternative terms like ‘long hauler’. We made this choice deliberately to concentrate on the official terminology recognized by the WHO. This decision was aimed at examining conversations specifically related to this officially endorsed term and understanding how it is interpreted and discussed in public forums.

### 5.2 Future directions

Future research could examine the persistence of user engagement by analyzing recurring posts to determine if repeated posters share distinct behaviors or concerns compared to general users. Investigating differences between personal and organizational accounts, as well as influencers versus non-influencers, could shed light on how various actors shape the conversation. Additionally, exploring associations between user bios and post content, including the use of emojis, could provide further insights into how identity and sentiment are expressed.

## 6 Conclusion

Online platforms play a role in community building, information dissemination, and in shaping public discourse around long COVID and its effects on individuals and societies. The public’s continued engagement and discourse on social media reflect the persistent and substantial burden of long COVID, necessitating concerted efforts to mitigate its effects and support affected individuals. The analysis has uncovered a multifaceted narrative surrounding long COVID and also underscores the need for continuing research, multidisciplinary collaboration, and comprehensive care strategies to address ongoing vaccine hesitancy and skepticism towards long COVID. Understanding the public’s concerns can guide targeted messaging and improve community outreach efforts, fostering trust in healthcare systems and promoting accurate information dissemination.

## Data Availability

The original contributions presented in the study are included in the article/Supplementary material, further inquiries can be directed to the corresponding authors.

## Supplementary material

SUPPLEMENTARY FIGURE S1 Temporal trends for the individual topics belonging to Theme 1, Theme 2 and Theme 3; normalized frequency indicates how often tweets on a specific topic occur at a given time, relative to the total number of tweets for the said topic across time.
