# Supplementary material for: Piecing together the narrative of #longcovid: an unsupervised deep learning of 1,354,889 X (formerly Twitter) posts from 2020 to 2023
Source: Front Public Health. 2024 Dec 16;12:1491087. doi: 10.3389/fpubh.2024.1491087 (PMC11683113; doi:10.3389/fpubh.2024.1491087)
Supplement: Supplementary file 1 [file Data_Sheet_1.docx]

**Supplementary Material**

Table S1. Sample tweets for the various topics, as per BERTopic modelling

| **Topic** | **Sample tweets** |
| --- | --- |
| Topic 1: Reports on long COVID | 1. So definitely not long COVID then? 2. The last one should have Long Covid. 3. The Other, Other 'Long Covid' 4. Long Remoan beats Long Covid every time! 5. If only long Covid werent a thing. 6. Damn. Long Covid is scary! 7. “Long COVID.” You can almost see the lightbulb flickering. Sooo close...Long Covid? What? 8. We don't know far more than we know. Long Covid Symptoms and Treatment 9. Dit is Long Covid 10. ‘Wait 2 weeks.’ (2 weeks pass) ‘Long covid!’ |
| Topic 2: Raising awareness about long COVID | 1. #LongCovid All kinds of stories 2. So much more to know about #longcovid 3. Assuming #LongCovid? I hope, you have support. 4. Wow. Nothing I can’t handle. Have you even HEARD of #LongCovid? Or are you choosing not to believe in it? 5. What does it show against #LongCovid? 6. Block the bot. #LongCovid is f***ing real; very serious. 7. Amid an "abyss of ignorance thats pervading the entire field" experts are trying to sort out #LongCovid. has the latest on where the data is pointing 8. Cant tell from the Twitter copy but this is also a story about #LongCovid. 9. Will be on at 9.40 am talking #LongCovid 10. #LongCovid could await him, like so many survivors. |
| Topic 3: Attributing long COVID to COVID-19 vaccines | 1. We cant I guess...but the side affects of long covid are absolutely horrendous for some, so its a trade off. Pretty much every vaccine for the last 60 years hasnt had any major or long lasting side effects so this is the same. 2. The myocarditis goes away, Long Covid apparently does not and there are 2.3 million people in King County so lets say 1 million vaccinated, so a transient side effect with a 33 in a million chance vs a possibility of a plastic tube shoved down my throat? Easy Choice. 3. ...full of mass organ failure, sepsis, heart problems &amp; severe body shutdown. #pfizer have also been commercialising this #vaccine, not for the good of "science" or your health &amp; have been involved in many drug scandals before. So, taking the Covid-V = Long Covid. 4. If you are young & healthy the mortality risk is not high, but "long covid" is still a significant problem. But....... it's not just about you. If enough people like you get vaccinated, herd immunity kicks in, your more vulnerable family & friends then become protected. 5. Isn't it true that people who are fully immunized have a very small risk of long covid? Or has that changed?? 6. Fascinating (once you can stretch an amateurs grey cells around the serious science) and positive article regarding a potential treatment in the absence of vaccine or in case of new variant escape... I wonder if this could also be helpful for long Covid? 7. Why don't we amend Long Covid apps to track vaccine side effects? 8. I am seeing some messaging that people who had Covid, particularly Long Covid cases, are being cautioned about taking the vaccine as some data is pointing to a flawed immune response as the primary cause. Is there a consensus yet on this? 9. Do we know if the vaccine saves lives or prevents Long Covid yet? 10. Not anymore. I’m actually better now than I was before getting sick. Took some work. Lost my trust in medical & big pharma. #TBIsurvivor #vaccineinjuries #LongCovid |
| Topic 4: Long term effects of long COVID in children | 1. This is consistent with a large burden of cases in kids, who are hospitalized at a much lower rate, but can still experience long-term consequences (long COVID). Wait for the hosp. rates 2 wks after Easter when the kids get together with Grandma & Grandpa & in churches 2. Long Covid in kids was one of my biggest worries (given low hospitalization rates), but we lacked a properly controlled study to know for sure what was going on. My fear of Long Covid in kids is pretty much erased with this finding. Always open for discussion. 3. Covid is lot more than only deaths (which are a horrendous aspect anyway). Long covid symptoms can be debilitating and children can have them too although with reduced incidence. Vaccines and physical barriers are our best bet. 4. 10 children died between 23rd Aug-26th Sept. 96 in total during the pandemic. Latest studies show healthy children are just as vulnerable. Then there's the long term risks e.g Long Covid, diabetes, brain damage (Biobank study), early onset dementia... 5. The “specialists and experts” of Long Covid in Mexico say that the disease in children (and adults) is not so bad that over time we recover, that we have to eat well, do rehabilitation, exercise and that's it. 6. Long Covid in children is very real and anyone who claims otherwise needs to get a grip on reality. 7. 11-15% of children will experience some form of long covid that's 5500 - 7500 kids in Texas alone who are likely to experience potentially permanent symptoms who knows how many more will have invisible organ damage that will impact them in the future 8. PVS in kids is nothing new obviously and needs to be taken seriously but the problem is that Long COVID covers a huge range of issues and time frames. Guess Sweden must have had at bare minimum 500k cases in U19 by now..what stats do we have on chronic outcomes &gt; 12 weeks? 9. Theres a misconception that kids dont get sick. Well now they do, and we know they might get long Covid. JP kids not immune. I hope (sadly) that teens death a few days ago will get parents really thinking. 10. Ive seen first hand long COVID in kids. Its devastating. |
| Topic 5: Importance of wearing masks as a prevention strategy from long COVID | 1. Also not sure if your aware of long covid but even if it doesnt kill you it can fuck you up for life. Is wearing a mask and social distancing that difficult for you? Would you prefer things get so out of hand were back on lockdown? 2. So true about long Covid , Ive gotten it twice. Thank goodness my husband has not gotten it. People here in my Town do not use mask at all. They think its over. Lol! I will and always mask everywhere I go. 3. The ability to contract it again and the increasing evidence of the shitshow of ongoing symptoms from long COVID? If you expect people to respect your decision not to wear a mask, you should be respectful the decision of others to wear one or request one be worn in their presence 4. This is the reason I mask. Im MUCH more afraid of the cascade of unintentional consequences of giving someone COVID than I am of getting it myself. What if someone gets long COVID, heart failure, diabetes or a stroke? Wearing a mask keeps the air AND my conscience clean. 5. Anyone remember the olden days when people thought that two-way masking didnt protect against #covid19, only the frail got sick and that #longcovid didnt exist? It appears that our top doctor is still living in the naive days of yesteryear. #BringBackMaskMandates 6. I have found reading the latest updates on Long Covid is a helpful motivator for me to keep masking. 7. Pajamas forever! Uniform of long covid 8. Duck the looks. Id rather feel conspicuous in a mask, than coughing and spluttering with long covid. :-) 9. Omicron is not “mild”. My family and I choose our “new normal” of masking, vaxing, hygiene. The fewer people wear masks, the more we'll be wearing our hi-fi masks. #Airgami #MaskUp 10. Sane Americans who do NOT want to get covid, or long covid, best mask up again. Sane Americans know that masking is easier than getting sick! |
| Topic 6: Public figures and COVID-19 | 1. Hmm It sounds like you just dont like him NSW had 200 cases recently from the Northern Beaches QT stuffup How many if those got Long COVID? Its no joke- I personally know people whove had it for 9 months. Dans rapid response minimised cases. Long COVID hits ~ 50% cases 2. He was anti-Vax and caught covid. Now he has long Covid. Its not the common code and theres reason to panic. 3. Either he is ill as you say - and well know how that turns out in the next few days, or he is well and just walked into a Petri dish. And even if he was ill but now well (unlikely) what about long covid? 4. If he recovers. Play the long Covid game. 5. With the widespread Long CoVID damage to come, the current overloading of the hospital system will seem like a fond memory!! Wonder how Morrison will deal with that? Escape to Hawaii permanently? Then hopefully get extradited. 6. Struggling from long covid, i heard. 7. has been struggling with Long Covid for 14 months so deserves a treat 8. They've apparently been a lot worse since he had Covid so looks like he has long covid. 9. but but he was supposed to have natural immunity! He can enjoy long Covid! 10. Oh my god elrich thinks he has long Covid? Were doomed. |
| Topic 7: Work absenteeism due to long COVID | 1. Its a shame that all those workers will have long Covid for the rest of their lives. 90% of them make less than 40K a year. Not even a livable wage . Politicians failed these workers families. #MonopoloyPower 2. Yep. And those affected early with long covid are being laid off now, with no extra financial help, just the below par minimum, so shoved into poverty 3. It's also one of the main drivers of 'long covid'.......especially if sick pay is being paid out 4. We had no option! How most of us would have loved a prolonged holiday on 80% rather than awful working conditions and virus exposure. The rate if long covid causing job loses in NHS is very high. Life changing 5. The same study found 15% of the entire US workforce shortage could be traced back to long COVID. Aside from all of this, the entire basis of the story is that there is insufficient testing, tracking, and prevention measures being taken by the government. 6. A reminder Sophie [because you have the intellect of a breeze-block] some of those public sector workers died during the course of their work, whilst others are disabled with #LongCovid What work do you do? other than spout bullsh*t 7. Good for the RMT. Decent sick cover; In the NHS if youve got long covid its 6 months full pay, 6 months half pay and then sacked. Even if you caught it on the frontline. The difference? Organise & act to preserve pay, terms, conditions. 8. No thought whatsoever for staff working in public facing environments either. We want to be back doing what we do best but we also dont want to end up passing the virus on to loved ones or coming down with long COVID. 9. As of February 2022, the syndrome was estimated to affect about 16 million adults in the U.S. and had forced between 2 million and 4 million Americans out of the workforce 10. Going in to month 30 of pandemic #1, 8% of US has long COVID. Per Brookings, labor force participation is only down 1% compared to 2019. So most of those people are working through it. |
| Topic 8: Cognitive issues associated with long COVID | 1. Long covid encephalopathy? 2. Key to understanding neuro/cognitive dysfunction during infection #LongCovid #MECFS 3. Long Covid: Significant evidence the virus can lead to brain-related neurological problems #NewsBreak 4. Long COVID triggers a hyperinflammatory state and 2 new studies show that long COVID has a notable impact on concentration and memory. 5. Long COVID PASC already is the third leading neurologic disorder in the United States, second only to tension-type headache and migraine. 6. Apparently this generation's big affliction is myalgic encephalomyelitis. Used to be known as chronic fatigue syndrome, but now sometimes attributed to Epstein Barr (mono) and Long Covid. Basically humans these days are blowing out their entire endocrine and nervous systems. 7. what about long covid and the damage that is doing? i have seen reports of people with long covid now developing alzheimers 8. Its funny how covid twitter is arguing about how valid the brain imaging Biobank study is as if its the only evidence of effect on brain. People with #LongCovid have been describing neuro symptoms for ages with multiple studies to characterise them. People before tests, folks! 9. Meanwhile... Profound neuroinflammation associated with long COVID 10. Exactly what I was thinking! Long COVID effects your memory. |
| Topic 9: Risk of long COVID with new variants of SARS-CoV-2 | 1. The survival rate with Omicron has been lower other than in the US where kids did badly with earlier variants. It's not just about surviving . The bigger issue has always been long Covid. 2. Huge rise in hospitalisation in under 12 from omicron. Also, no idea of long term implications given micro clot issues. Plus long covid numbers are a concern. Which makes it very risky. 3. Approx 1.3 million Canadians estimated to have Long COVID from Omicron. Mild my ass. 4. Erm, if you prevent the short version of COVID, you prevent the longer one. Breakthrough still can result in long COVID...of course its less common in vaccinated population! We won't know about severity of omicron for weeks yet. Delta is still a massive problem, regardless. 5. Thanks for the link to the abstract, Im a realist and there is no stopping Omicron with masking or lockdowns. Yes multiple Reinfections are what #endemic means. Fingers crossed they work on long COVID treatment. 6. “Is there long covid w/ #Omnicron?,” ask “It's too early to say...In South Africa, a lot of people got infected with Omnicron but a good many had been vaccinated,” says Global Incident Manager for COVID19 Incident Management System Team 7. And then there's #LongCovid I didn't see it mentioned an only news channel that, despite a statistically lower risk from Omicron than Delta there were more people with LC from Omicron than Delta because of the number of cases! Yet new variant is recombined Delta and Omicron! 8. No data on this that Ive seen. And confounded by progressively more immunity built over time and lower incidence of Long Covid from Omicron lineages than prior major variants (which may be interrelated) 9. Early data suggest the omicron variant is similar in severity to the delta strain. What is certain, is that letting the omicron variant spread through Queensland will result in the deaths of a lot of people. Countless more will be left with long COVID. 10. Thats dangerously wrong information. Omicron still kills, and causes Long Covid. It is still one of the most dangerous viruses. You should never get infected, because it might ruin your life, or kill you. And immunity will only last a few months, then you can get infected again. |
| Topic 10: Denying the existence of long COVID | 1. Sounds like she doesnt have long Covid and is denying people whove been vaccinated can get it. Thats not true. Its misinformation. Shes spreading it. If she wont protect people by providing accurate information, I hope she has the opportunity to serve as a cautionary tale. 2. Most of these cases are Vaccine Side Effects do your proper research And Sylvana CLAIMS to have Long Covid ... so she can lay in her bed watch Netflix while Dutch Blacks are Starving & Gentrified. Empty Bij1 Parliament seat at every meaningful debate BLM Grift. 3. Long covid has been grossly over estimated. She needs to read up to date journals. Or stick a mask on and lie down somewhere quiet...give the rest of us a bit of peace 4. She's a denier and I'm a healthcare worker who got long covid, either from her bringing her granddaughter to Xmas with a 'cold' or from an a-hole coworker. Either way, her utter glee at lying to everyone for Xmas got me all the way done with her. We'll see. #IHaveBoundariesNow 5. Yep, and shes counting death as the only possible outcome, totally ignoring Long Covid - organ damage etc which IS impacting the young and children despite the narrative of experts like her and that Schofield person. 6. Perhaps and as I decent human she might have an insight into my dx of Long Covid..nah 7. Idiot, Julia has had it, along with the rest of her family. She even has long Covid symptoms (loss of taste). Julia doesnt however think making children suffer helps anyone because shes not stupid 8. She is not a scientist just got some screenshots. I can do the same, look at this: IQ deceased for kids had long Covid. A dumber generation? 9. Long COVID is just because you don't do enough exercise 10. If only she understood this. And I'm not sure what that has to do with my point. She won't get long covid from people “wishing” anything. |
| Topic 11: Relationship between long COVID and chronic fatigue syndrome | 1. The ME-ICC involve a complex set of menus and criteria a patient must meet to be identified as having ME. Incidentally, one of the criteria of ME-ICC is PESE, so the current terminology being adopted by patients with Long Covid only 2. Possibly because all of these conditions can be triggered by viruses including FND, though through lack of awareness it carries a psychogenic stigma that ME/CFS and Long Covid want to distance themselves from. 3. Finally, the SO CALLED medical professionals are attempting to do something about cfs; Fybromyalgia; and even Long Covid symptoms... Better late than never!!! Or is it just lip service to pamper the Masses??? 4. Well to throw into that mix I have Fibromyalgia which has very similar symptoms to both Long Covid and MECFS. I think each diagnosis stands on its own accord but were all in this together. Chronic illness like these need to work together, some treatments 5. One of my kids has CFS/ME - long covid is very very similar. It is not something you want to mess with. Get kids vaccinated ASAP. You can catch ueducation 6. #longhauler who had Covid in November and developed severe #mecfs after. Me/cfs research can deliver solutions for the #longcovid pathology. We need solutions!! #pwME 7. MUST READ Large Long COVID Study and Major Media Articles Underscore Link to #ME/CFS - Plus Countdown for the NIH - Health Rising 8. A small % of #LongCovid, as with any serious virus, may develop ME. Still q. early to tell - although a small no. of those who exercised through Covids PVF have been presenting w. CFS. Covid-19 is unique - crucially its all taking longer (so talk of ME still a bit previous). 9. I've been experimenting on myself for 30 years & have been seen by so many dismissive condescending doctors who don't know the first thing about CFS (while I'm a walking database). ME/MCAS/hEDS left me vulnerable to #LongCovid and now like so many, i'm living through THAT... 10. For too long ME/CFS has been ignored and has been underfunded. Had they been supported “Long Covid” patients would be helped better. |


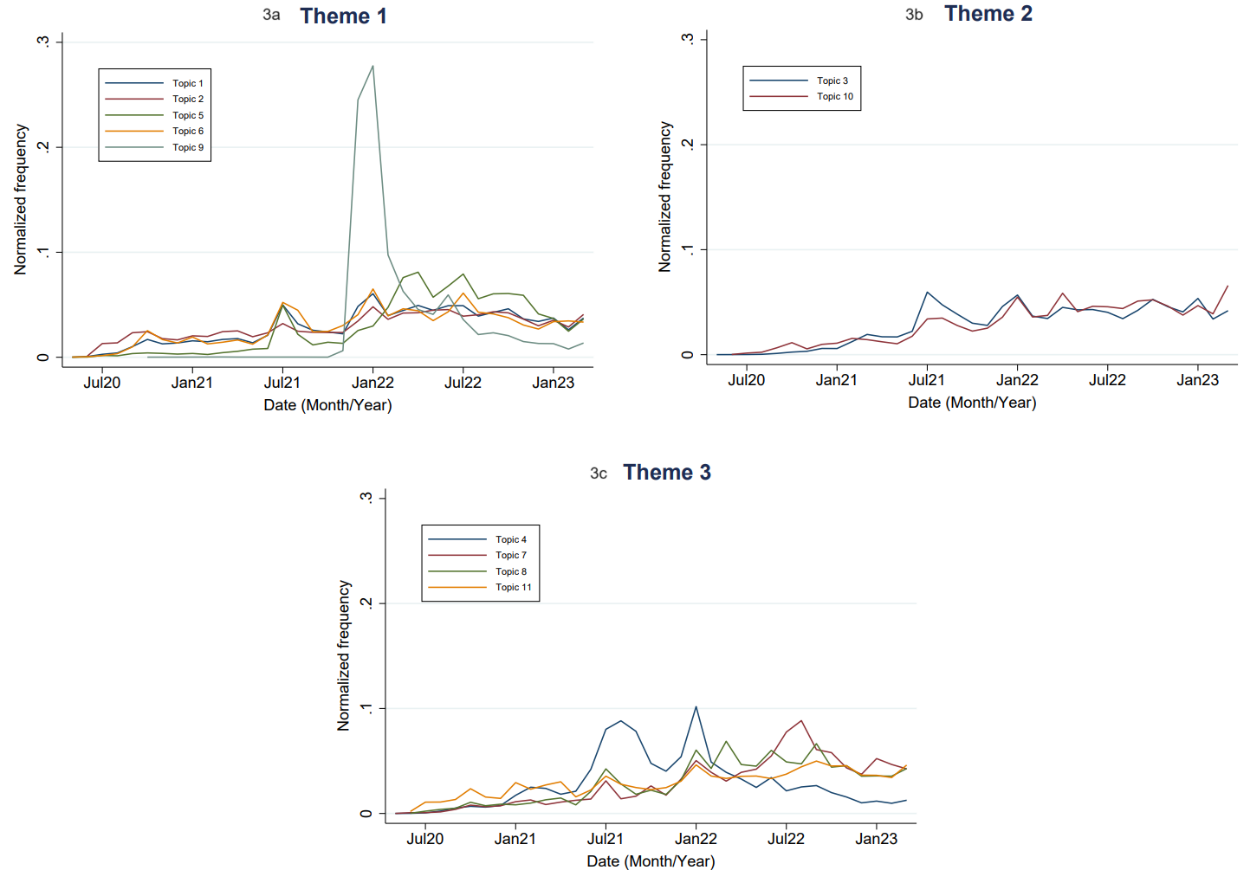


Fig. S1. Temporal trends for the individual topics belonging to Theme 1 (Fig S1a), Theme 2 (Fig S1b) and Theme 3 (Fig S1c); normalized frequency indicates how often tweets on a specific topic occur relative to the total number of tweets in a given time period
