# Supplementary figures and images for: Piecing together the narrative of #longcovid: an unsupervised deep learning of 1,354,889 X (formerly Twitter) posts from 2020 to 2023
Source: Front Public Health. 2024 Dec 16;12:1491087. doi: 10.3389/fpubh.2024.1491087 (PMC11683113; doi:10.3389/fpubh.2024.1491087)

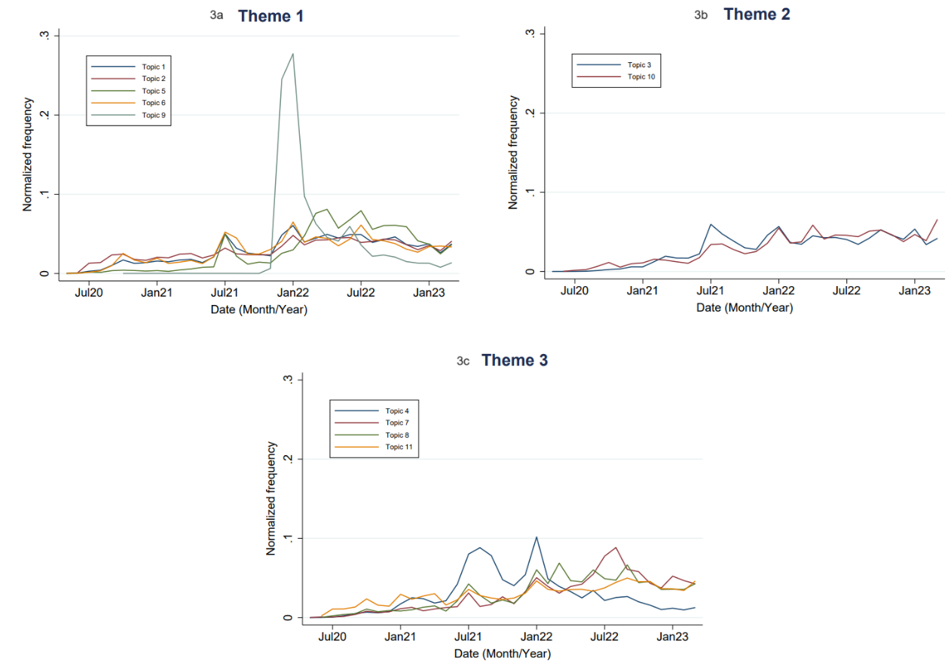

Supplement: SUPPLEMENTARY FIGURE S1 — Temporal trends for the individual topics belonging to Theme 1, Theme 2 and Theme 3; normalized frequency indicates how often tweets on a specific topic occur at a given time, relative to the total number of tweets for the said topic across time. [file Image_1.PNG]
